# *Mertk* ^*+*^ Liver Sinusoidal Endothelial Cells Negatively Regulate PINK1 Related Mitophagy and Accelerate MASH

**DOI:** 10.1002/iid3.70256

**Published:** 2025-09-18

**Authors:** Yu‐Xuan Gao, Zhong Weng, Long Tang, Ming‐Yi Xu, Sheng‐Zheng Luo

**Affiliations:** ^1^ Department of Gastroenterology, Shanghai East Hospital, School of Medicine Tongji University Shanghai China; ^2^ Ningde Clinical Medical College of Fujian Medical University Ningde Fujian China; ^3^ Ningde Municipal Hospital of Ningde Normal University Ningde Fujian China; ^4^ Department of Gastroenterology, Shanghai General Hospital Shanghai Jiaotong University, School of Medicine Shanghai China

**Keywords:** extracellular regulated protein kinase (ERK), liver sinusoidal endothelial cell (LSEC), Mer tyrosine kinase (Mertk), metabolic dysfunction‐associated steatohepatitis (MASH), mitophagy, PTEN‐induced putative kinase 1 (PINK1)

## Abstract

**Background:**

Mer tyrosine kinase (*Mertk*) regulating mitochondrial function of liver sinusoidal endothelial cells (LSECs) in metabolic dysfunction‐associated steatohepatitis (MASH) remains unclear.

**Methods:**

*Mertk/p‐Mertk*, PINK1, and ERK/p‐ERK expression in steatotic LSECs and livers of MASH mice were studied. Mitochondrial functions were assessed via immunofluorescence, Western blot, and qPCR. *C‐Kit* ^+^‐bone marrow cells (BMCs)^*sh‐Mertk*^ were bone marrow transplanted (BMT) to MASH mice to evaluate its effect.

**Results:**

Ov‐*Mertk* would markedly stimulate ERK, and ERK further suppress downstream PINK1. Higher levels of *Mertk/p‐Mertk* and lower levels of PINK1 were confirmed in steatotic LSECs and MASH mice livers. Steatotic LSECs^*sh‐Mertk*^ exhibited intact mitophagy, integral mitochondrial membrane potential, reduced reactive oxygen productions and upregulation of the PINK1 pathway. BMT of *C‐Kit* ^+^‐BMCs^*sh‐Mertk*^ could equivalently protect mitochondrial functions and ameliorate lipid accumulation in MASH mice.

**Conclusion:**

*Mertk* negatively regulates PINK1‐mediated mitophagy in LSECs through the p‐ERK signaling pathway, thereby accelerating MASH progression. Therefore, LSECs deficient of *Mertk* should be a novel therapy for reversing PINK1‐related mitophagy and MASH.

## 1 Introduction

MASLD (metabolic dysfunction‐associated steatotic liver disease) and MASH (metabolic dysfunction‐associated steatohepatitis), formerly also known as NAFLD and NASH, already become a heavy economic and social burden, and an important cause of the end stage liver disease [1]. The hallmark of MASLD is > 5% fat accumulation in the liver, this steatosis would progress to subsequent inflammatory MASH, followed by advanced fibrosis. The molecular mechanisms driving MASLD pathogenesis include dysregulation of lipid metabolism, chronic inflammation, oxidative stress, mitochondrial dysfunction, and gut microbiota alterations [2]. MASLD/MASH, so‐called chronic low‐grade inflammation, in which spleen plays a key role being the crossroad between inflammation and immunity [3]. Recently studies suggested that changes in cytokine production by splenic lymphocytes of MASLD mice are indicative of altered immune functions through lymphocytes, monocytes, T cells, B cells and natural killer cells, which might contribute to MASLD [3].

Among various factors, mitochondrial dysfunction plays a key role in the pathogenesis of MASH. In MASH, mitochondrial dysfunction could impair fatty acid oxidation and lipid metabolism, lead to impaired ATP production and cellular energy deficiency, accumulate reactive oxygen species (ROS) and cause hepatocyte death [4]. Moreover, dysfunctional mitochondria would trigger inflammatory responses through various pathways, including damage‐associated molecular pattern release and inflammatory signaling activation, which contribute to liver fibrosis and cirrhosis progression [5]. Increased mitochondrial impair, suggested loss of the mitochondrial quality control pathway known as mitophagy that selectively degrades damaged mitochondria via the autophagosomal or lysosomal pathway. Undamatla et al proved that loss of mitophagy occurred early in the pathogenesis of MASH through PINK1 (PTEN‐induced putative kinase 1)‐Parkin pathway [6]. We also clarified MASH could lead to damage of PINK1 related mitophagy and aggravate liver fibrosis [7].

Among nonparenchymal cells (NPCs) population, LSECs (liver sinusoidal endothelial cells) are the major in the liver. In MASH, lipotoxic LSECs would stimulate abnormal signaling, initiate structural and functional alterations, and manifest pro‐inflammatory phenotype [8]. *Mertk* (Mer tyrosine kinase), one of Tyro‐Axl‐Mer (TAM) family, is an important receptor for the clearance of apoptotic cells, as efferocytosis. Wu et al found that deficiency of *Mertk* could aggravate atherosclerosis through upregulation of dysfunction and decreasing endothelial efferocytosis in aortic endothelial cells (ECs) [9]. Our recent study identified that *Mertk* promotes capillarization and angiogenetic properties of LSECs in MASH, thus knocking‐down *Mertk* of LSECs could alleviate endotheliopathy and MASH (unpublished data). Thus, how *Mertk* regulates mitochondrial function of LSECs and their alteration in MASH remains unknown.

Herein, we showed that steatotic LSECs expressed high level of *Mertk*; while *Mertk* in turn inhibited PINK1 related mitophagy in vitro and in vivo. In contrast, transplanting protective *Mertk* deficient *C‐Kit* ^+^‐bone marrow cells (BMCs, also representing bone marrow–derived endothelial progenitor cells [10]) would reverse PINK1 mediated mitophagy and lipidosis in MASH mice. Our study innovatively identify a potential role of LSECs regulation of mitochondrial functions through *Mertk*/p‐ERK/PINK1 pathway in MASH. Therefore, transplanting of LSECs deficient of Mertk maybe a novel therapeutic strategy for managing MASH.

## 2 Materials and Methods

### 2.1 Mouse Model

C57BL/6 mice (8‐week‐old, Shanghai Laboratory Animal Co. Ltd., Shanghai, China) randomized in 4 groups (*n* = 5 per group): (1) The control group, which was fed a low‐fat diet for 8 weeks; (2) The MCD group, which was fed a MCD (deficient in methionine and choline diet of 40% carbohydrate, 10% fat) for 8 weeks; (3) The BMT (bone marrow transplanting)‐shNC group, which received a MCD for 8 weeks, and transplanted of *C‐Kit* ^+^‐primary bone marrow cells (*C‐Kit* ^+^‐pBMCs) expressing a short hairpin nontargeting control (sh‐NC) in the last 2 weeks; and (4) the BMT‐*shMertk* group, which received a MCD for 8 weeks, and transplanted of *C‐Kit* ^+^‐pBMCs expressing a shRNA targeting *Mertk* (*sh‐Mertk*) in the last 2 weeks.

Before BMT, recipient mice were subjected to lethal irritation. The donor *C‐Kit* ^+^‐pBMCs [11] transfected with *sh‐Mertk* or sh‐NC, and then 5 × 10^6^ cells per recipient mouse were transplanted via tail vein. All the BMT mice were euthanized 2 weeks postprocedure [12].

The animal study was approved by the Institutional Animal Care and Use Committee of Shanghai East Hospital.

### 2.2 Histological Staining

Fixed overnight with 10% formalin, mouse liver tissues were paraffin‐embedded and sectioned into 4 µm thickness. Sections were stained with hematoxylin‐eosin (H&E), Masson's trichrome and oil red O (ORO). The Leica microscope was used to observe the images. Quantitative histomorphometric analysis was conducted utilizing Image J 1.8.0 software. NASH activity score (NAS) was performed to assess pathological changes and liver tissue inflammation.

### 2.3 Mouse Primary Cells

The pLSECs (primary LSECs) suspension was received after warm collagenase perfusion (Roche, Basel, Switzerland) and centrifuged to obtain the NPC fraction. Then, the suspension was centrifuged through Percoll gradients (Yeasen Biotech, Shanghai, China) to obtain pLSECs [13]. The pLSECs were cultured in ECM medium (ScienCell, CA, USA) accompany with 5% FBS (Invitrogen, CA, USA) along with antibiotics and antimycotics (Sigma‐Aldrich, MO, USA).

Isolation of pBMCs was referred to previously reported method [14]. Following euthanasia, the femurs and tibias of mice (8‐weeks, C57BL/6, male, *n* = 5) were dissected, and marrow content was flushed using a syringe and buffer. The flush solution underwent filtration, and the resulting solution was centrifuged, yielding pellets of pBMCs. Then, magnetic activated cell sorting (MACS; Miltenyi Biotec, Cologne, Germany) was employed to obtain donor *C‐Kit* ^+^‐pBMCs. These pBMCs were cultured in DMEM (HyClone, UT, USA) with 10% FBS and antibiotics. The typical yield generally ranges from 1 × 10^7^ to 1.2 × 10^7^ progenitor cells per mouse.

### 2.4 Cell Line

TMNK‐1 cells (human LSECs) were cultured in DMEM with 10% FBS. To mimic a lipotoxic environment, 200 μM palmitic acid (PA; Sigma‐Aldrich) was pretreated, or 3% BSA (bovine serum albumin; Sigma‐Aldrich) as the control. TMNK‐1 cells were also treated with DMSO (1%, Biosharp, Beijing, China) and SCH772984 [300 nM, a selective extracellular regulated protein kinase (ERK) phosphorylation inhibitor, MCE, Shanghai, China].

### 2.5 Cell Transfection

For Mertk knockdown, *sh‐Mertk* (5′‐GCTCAATCAGTGTACCTAATA‐3′) or sh‐NC were inserted into the pLKO.1‐puro plasmid. For *Mertk* overexpression, a full length *Mertk* was cloned into the pCMV‐3×FLAG‐SV40‐Neo plasmid (ov‐*Mertk*), and an empty vector as control (Vigene Biosciences, Shandong, China). Transduction effectiveness was investigated after 48 h through qPCR.

### 2.6 MitoSOX and MitoTracker Red CMXRos

MitoSOX Red (Invitrogen), a mitochondrial superoxide indicator, was used to visualize mitochondrial reactive oxygen species (ROS). MitoTracker Red CMXRos Kit (Invitrogen) was used to assess mitochondrial membrane potential (MMP). Leica fluorescence microscope was used. For quantitative analysis, ≥ 100 cells per condition were analyzed from three independent experiments, with random sampling of 5 fields per replicate using ImageJ under blinded conditions.

### 2.7 PCR

QPCR was run using a SYBR Green PCR Master mix on a QuantStudio 5 (Applied Biosystems, CA, USA). List of the primers could be seen (Sangon Biotech, Shanghai, China, Supporting Information S1: Table S1).

### 2.8 Western Blot

Extracted proteins were separated by SDS‐PAGE (10%) and transferred to PVDF membranes (Millipore, Billerica, MA, USA). Membranes were blocked with 5% nonfat milk for 1 h, and then incubated with the primary antibody at 4°C. Membranes were washed the next day and incubated with secondary HRP‐conjugated antibodies for 2 h. The primary and secondary antibodies used are shown in Supporting Information S1: Table S2.

### 2.9 Immunofluorescence (IF) Assay

Sections of cell or livers were incubated overnight with primary antibody at 1:500 ~ 1000 dilution, washed, and then incubated with 1:10000 dilution of secondary antibody for 1 h (Supporting Information S1: Table S3). DAPI was used to label nuclei, and images were examined using the Leica fluorescence microscope. All immunostaining quantifications included 3 biological replicates, with ≥ 100 cells scored per replicate across randomly selected fields.

### 2.10 Statistical Analysis

Data were represented as mean ± SD, with at least three times each experiment. An unpaired *t*‐test was used for comparisons between two groups. All statistical analyses were performed using GraphPad Prism 11. A *p*‐value less than 0.05 was considered statistically significant. The statistical significance (*p* < 0.05) was assessed.

## 3 Results

### 3.1 *Mertk* Negatively Regulates PINK1 Through p‐ERK Signaling in LSECs in MASH

**Figure 1:**
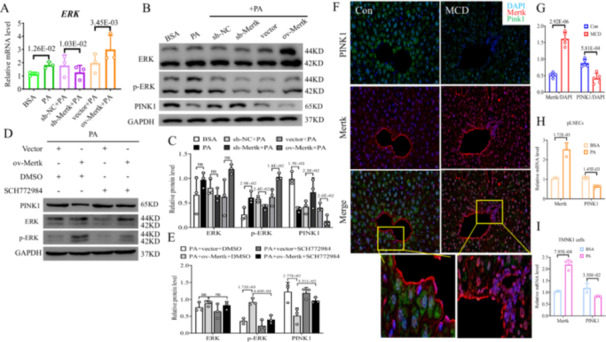
*Mertk* negatively regulates PINK1 through p‐ERK in LSECs and the expression of them in MASH. TMNK‐1 cells were divided into 6 groups (BSA, PA, sh‐NC + PA, sh‐*Mertk* + PA, vector+PA and ov‐*Mertk* + PA): (A) The mRNA levels of ERK were examined in six groups by PCR; (B, C) ERK/p‐ERK and PINK1 proteins in six groups were determined via western blot analysis. TMNK‐1 cells were divided into four groups (PA+vector+DMSO, PA+ov‐*Mertk* + DMSO, PA+vector+SCH772984, PA+ov‐*Mertk* + SCH772984): (D, E) PINK1 and ERK/p‐ERK proteins in four groups were examined by western blot analysis. (F, G) IF staining of *Mertk* (red)/PINK1 (green)/DAPI (blue) and quantification were shown in two groups of mice (Con and MCD, each group *n* = 5, scale bars = 20 μm). And the areas of yellow frame were further enlarged. (H, I) *Mertk* and PINK1 mRNAs in BSA/PA treated pLSECs and TMNK‐1 cells were examined via qPCR. The *p*‐value indicates a significant difference compared to BSA/sh‐NC + PA/vector+PA/vector+PA + DMSO/ov‐*Mertk* + PA + DMSO treated TMNK‐1 cells; control mice; BSA treated pLSECs/TMNK‐1 cells.

*Mertk* was identified to regulate the ERK signaling pathway [15]. Afterwards, inhibition of ERK would stimulate PINK1 related mitophagy pathway [16]. PINK1, a sensor of damaged mitochondria, plays a key role in regulating mitophagy in MASH [17]. Hence, we focused on whether *Mertk*/ERK/PINK1 pathway exists and alters in LSECs in MASH. TMNK‐1 cells were divided to 6 groups including BSA, PA (palmitic acid), sh‐NC + PA, sh‐*Mertk* + PA, vector+PA, and ov‐*Mertk* + PA. Sh‐*Mertk* + PA obviously downregluated *Mertk*, whereas as expected PA slightly upregulated and ov‐*Mertk* + PA significantly upregulated *Mertk* in TMNK‐1 cells (Supporting Information S1: Figure S1A‐S1B). In the ov‐*Mertk* + PA and PA group, expressions of ERK/p‐ERK mRNA and proteins were upregulated compared to each control group (Figure 1A–C). However, in the sh‐*Mertk* + PA group, expressions of them were downregulated compared to sh‐NC + PA group (Figure 1A–C). Thus, *Mertk* is confirmed to activate the p‐ERK. To understand the effect of *Mertk* in inhibiting PINK1 induced by ERK, SCH772984 (an ERK inhibitor) was used in vitro to block the p‐ERK. The decrease of PINK1 levels and augment of ERK phosphorylation because of *Mertk* overexpression, was totally blocked after ERK inhibitor administration (*p* < 0.05; Figure 1D,E). As expected, the protein levels of PINK1 were significantly decreased in PA or ov‐*Mertk* + PA cells; in contrast, it was dramatically increased in sh‐*Mertk* + PA cells (*p* < 0.05; Figure 1B,C). In summary, our data suggest that *Mertk* inhibits PINK1 by activating the p‐ERK signaling in LSECs in MASH.

### 3.2 Expressions of Interacting *Mertk* and PINK1 in MASH

To investigate the expression of *Mertk* and PINK1 in MASH in vivo, we established MCD‐fed MASH mice. Substantial steatohepatitis, lipid accumulation and fibrosis were revealed significantly worse in the livers of MCD mice in contrast to control mice (Figure 4A–D). Additionally, IF staining demonstrated that *Mertk* ^*+*^ cells (red IF) were markedly augmented whereas PINK1^+^ cells (green IF) was reduced in the livers of MCD mice (MCD vs. Con: *Mertk* with a 3.2‐fold increase; PINK1 with a 0.5‐fold decrease; *p* < 0.05; Figure 1F,G). To validate the alterations of *Mertk* and PINK1 in MASH in vitro, the expression levels of pLSECs and TMNK‐1 cells subjected to BSA or PA 200 μM 24 h were detected by qPCR. PA treatment significantly upregulated *Mertk* levels while downregulated PINK1 levels compared to BSA treatment in both pLSCEs and TMNK‐1 cells (*p* < 0.05; Figure 1H,I). Collectively, these findings indicate that lipotoxic environment would influence the expressions of *Mertk* and PINK1, highlighting their potential role in hepatic steatosis and metabolic stress.

### 3.3 *Mertk* Inhibits PINK1‐Related Mitophagy in LSECs of MASH

**Figure 2:**
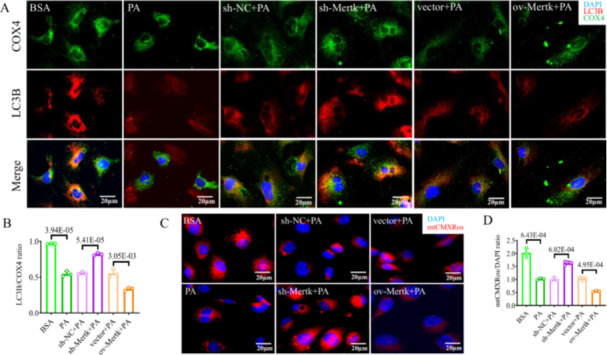
*Mertk* could impair mitophagy and enhance mitochondrial depolarization in LSECs. IF images and quantification of (A, B) LC3B (red IF), COX4 (green IF), DAPI (blue IF); (C, D) mtCMXRos (red IF)/DAPI (blue IF) in six groups. (A–D) Scale bars = 20 μm. The *p*‐value indicates a significant difference compared to the BSA/sh‐NC + PA/vector+PA group.

**Figure 3:**
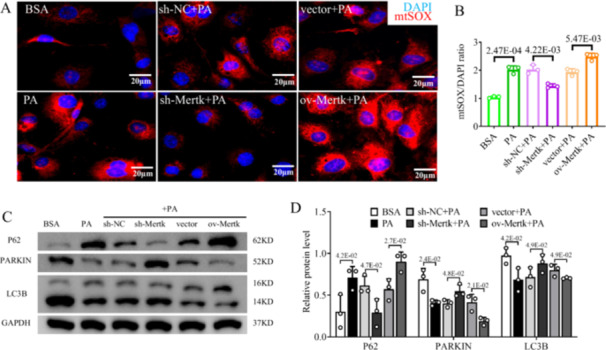
*Mertk* could suppress PINK1‐mediated mitophagy and induce mitochondrial oxidative damage in LSECs. (A, B) IF staining and quantification of mtSOX (red IF)/DAPI (blue IF) in six groups. (C, D) PARKIN, LC3B and P62 proteins in six groups were determined via western blot analysis. (A, B) Scale bars = 20 μm. The *p*‐value indicates a significant difference compared to the BSA/sh‐NC + PA/vector+PA group.

Mitophagy, a mitochondrial quality control mechanism that removes dysfunctional mitochondria, is inhibited during the development of MASH [6]. To evaluate the effect of *Mertk* on mitophagy in LSECs, costaining for LC3B (light chain 3B, a marker of autophagy; red IF) and COX4 (cytochrome c oxidase subunit 4, a marker of mitochondria; green IF) was performed via IF. Treatment with PA and ov‐*Mertk* + PA decreased the colocalization of mitochondrial autophagy (orange IF), suggesting mitophagy deficiency; however, sh‐*Mertk* + PA treatment increased this colocalization (0.56‐fold decrease in the PA vs. BSA group; 0.61‐fold decrease in ov‐*Mertk* + PA vs. vector+PA group; 1.48‐fold increase in sh‐*Mertk* + PA vs. sh‐NC + PA group; *p* < 0.05; Figure 2A,B). Mitophagy disturbance occurs along with the loss of the MMP and excessive mitochondrial ROS production [18, 19]. PA and ov‐*Mertk* + PA treatment could induce a decrease in the MMP, whereas sh‐*Mertk* + PA treatment ameliorated mitochondrial depolarization, as shown by IF staining of mtCMXRos (MitoCMXRos) red (PA vs. BSA group: 0.50‐fold; ov‐*Mertk* + PA vs. vector+PA group: 0.54‐fold; sh‐*Mertk* + PA vs. sh‐NC + PA group: 1.63‐fold; *p* < 0.05; Figure 2C,D). Additionally, IF staining of mtSOX (MitoSOX) red revealed that PA and ov‐*Mertk* + PA treatment could increase mitochondrial ROS production, which was alleviated by sh‐*Mertk* + PA treatment (PA vs. BSA group: 1.94‐fold; ov‐*Mertk* + PA vs. vector+PA group: 1.28‐fold; sh‐*Mertk* + PA vs. sh‐NC + PA group: 0.71‐fold; *p* < 0.05; Figure 3A,B). PINK1/Parkin‐dependent mitophagy signaling pathway is shown to play key role in MASH [7]. We also detected proteins involved in the PINK1/Parkin axis in 6 groups of LSECs. The levels of PINK1, PARKIN and LC3B proteins were lower, but the protein levels of P62 proteins were greater in PA and ov‐*Mertk* + PA groups than in their controls, suggesting the inhibition of mitophagy (*p* < 0.05; Figure 1B/3C,D). In contrast, sh‐*Mertk* + PA treatment markedly rescued mitophagy by increasing PINK1, PARKIN, and LC3B proteins and decreasing P62 proteins compared to the controls (*p* < 0.05; Figures 1B/3C,D). Taken together, these results suggest that *Mertk* inhibits the PINK1/Parkin‐related mitophagy in LSECs in MASH.

### 3.4 Transplanting of *C‐Kit* ^+^‐BMCs^*sh‐Mertk*^ Could Reverse Hepatic PINK1‐Regulated Mitophagy and Liposis in MASH Mice

**Figure 4:**
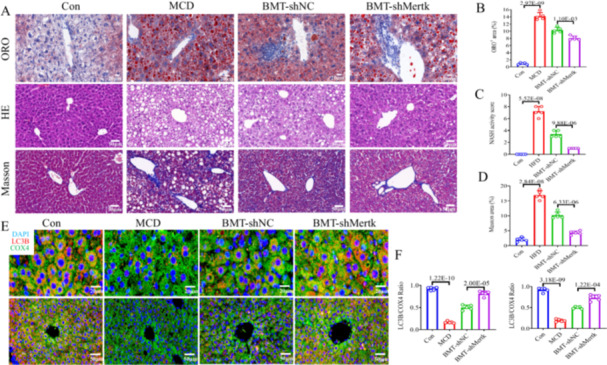
BMT of *C‐Kit* ^+^‐BMCs^*sh‐Mertk*^ could alleviate mitophagy and MASH in mice. Four mice groups included Con (chow diet‐fed mice), MCD (MCD‐fed mice), BMT‐shNC (BMT of *C‐Kit* ^+^‐BMCs^sh‐NC^ to MCD‐fed mice), and BMT‐*shMertk* (BMT of *C‐Kit* ^+^‐BMCs^sh‐Mertk^ to MCD‐fed mice), each group *n* = 5. (A–D) Images and quantitative analysis of ORO, H&E, and Masson staining of livers from the 4 groups (scale bars: ORO 20 μm, H&E/Masson 50 μm). (E, F) IF images and quantification of LC3B (red IF), COX4 (green IF), and DAPI (blue IF) in the parenchymal and portal areas (scale bars: parenchymal areas 20 μm, portal areas 50 μm). The *p*‐value indicates a significant difference compared to the Con/BMT‐shNC group.

**Figure 5:**
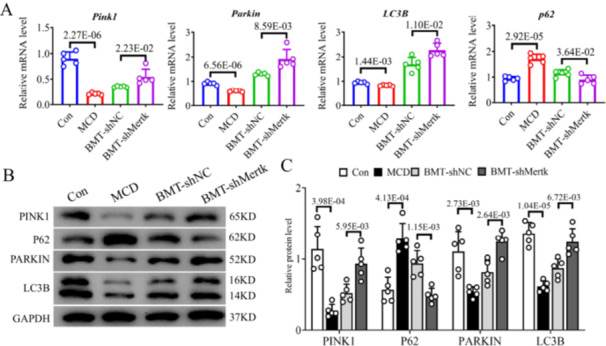
BMT of *C‐Kit* ^+^‐BMCs^*sh‐Mertk*^ could ameliorate PINK1‐mediated mitophagy in MASH mice. Expressions of (A) mRNA and (B, C) protein of PINK1, PARKIN, LC3B and P62 in four groups were determined via qPCR and western blot analysis. The *p*‐value indicates a significant difference compared to the Con/BMT‐shNC group.

To explore a therapeutic effect on suppression of *Mertk* in LSECs in vivo, *C‐Kit* ^+^‐BMCs^*sh‐Mertk*^ were transplanted through bone marrow to MASH mice. *C‐Kit* ^+^‐BMCs transfected with sh‐NC or sh‐*Mertk* were transplanted to MCD‐fed mice after lethal X‐ray irradiation; thus named BMT‐shNC and BMT‐*shMertk* groups. Obvious upregulation of *Mertk* mRNA and proteins was seen in MCD‐fed mice compared to control mice; but noticeable downregulation of *Mertk* mRNA and proteins was detected in BMT‐*shMertk* mice compared to BMT‐shNC mice (Figure S2A‐S2B). Steatohepatitis, lipid deposition and fibrosis were obviously aggravated in the MCD‐fed mice compared with control mice (Figure 4A–D). However, transplanting *C‐Kit* ^+^‐BMCs^*sh‐Mertk*^ markedly reduced above appearance compared to transplanting *C‐Kit* ^+^‐BMCs^sh‐NC^ into MCD‐fed mice (Figure 4A–D). We further assessed mitophagy in MCD‐fed mice transplanted with different *C‐Kit* ^+^‐BMCs. IF staining of COX4 (green IF) and LC3B (red IF) revealed that the number of cells with colocalization of orange among the liver parenchyma and portal area was lower in MASH mice than control mice (*p* < 0.001; Figure 4E,F). Furthermore, BMT‐*shMertk* mice had brighter orange IFs in those areas than did BMT‐shNC mice (*p* < 0.001; Figure 4E,F). Stimulation of PINK1/Parkin‐mediated pathway exacerbates mitophagy. A decreasing gene and protein expressions of PINK1, Parkin and LC3B, but increasing expressions of p62 were found in MCD mice versus control mice (Figure 5A–C). BMT of *C‐Kit* ^+^‐BMCs^*sh‐Mertk*^ exhibited an increase in PINK1/Parkin pathway‐related factors compared to BMT of *C‐Kit* ^+^‐BMCs^sh‐NC^ (Figure 5A–C). These results suggest that inhibiting Mertk in BMCs could effectively resuscitate PINK1/Parkin‐mediated mitophagy and MASH in vivo.

## 4 Discussion

A growing evidence implicates LSEC dysfunction in MASH. Khoury et al identified GSK3 would induce myeloid cell associated liver inflammation and LSEC endotheliopathy, while GSK3 inhibitors had therapeutic efficacy in murine MASH [20]. Wang et al indicated that USP9X delivered via extracellular vesicles from mesenchymal stem cells could inhibit LSEC angiogenesis and MASH related liver fibrosis [21].

LSECs are key actors in cellular crosstalk. The endothelium generates a connection line with hepatocytes in liver. Metabolism of ECs depends on their mitochondrial function responding to flow change [22]. Cheng et al found that Carma3 localized more in mitochondria of LSECs, and autoimmune hepatitis could promote mitochondrial and cellular impair in Carma3^‐^ LSECs [23]. After warm ischemia, mitochondria of LSECs was revealed with morphological abnormalities, ribosome or endoplasmic reticulum reduction [24]. But the potential role and molecular signaling pathway participated in mitochondrial function of LSECs is still indistinct in MASH.

*Mertk* mainly expresses in macrophages and immature dendritic cells, and also expresses in ECs [25]. Happonen et al. demonstrated that *Mertk* is a key regulator of angiogenesis and blood‐brain barrier integrity in the mature vasculature [26]. Li et al. reported that *Mertk* knocking‐down could alter adherens junction structure and decrease junction‐related protein levels and basal Rac1 activity in ECs [27]. Xu et al. established mitochondrial *Mertk* ^*hi*^ macrophages and injected to the acute spinal cord injury region; and then phagocytosis enhanced, lipidosis reduced, mitochondrial function improved, and inflammation alleviated in macrophages [28]. Deficiency in the *Mertk* of cardiac macrophages would lead to less myocardial mitochondrial elimination and autophagy, but more abnormal mitochondria and ventricular function [29]. The mechanism about how *Mertk* of LSECs affects mitochondrial function in MASH is still unclear. We proved overexpressing *Mertk* could obviously stimulate ERK, and then ERK further inhibit downstream PINK1. The expressions of *Mertk* were increased but expressions of PINK1 were opposite in MASH in vivo and in vitro. It is well recognized that PINK1 mediated mitophagy pathway plays an important role in MASH [7, 17]. Our results first define that *Mertk* negatively regulates PINK1 by activating the p‐ERK signaling in LSECs in MASH. The present study provides a valuable insight into the potential role of LSECs regulation of mitochondrial functions through *Mertk*/p‐ERK/PINK1 pathway in MASH.

BMCs have been implicated as a modifiers of vascular growth either directly by transdifferentiation into ECs or indirectly through growth factor release [30]. Shabani et al. [31] elucidated the heterogeneity of BM‐derived ECs in the heart and their response to repetitive ischemia, laying the groundwork for targeting specific subpopulations for therapeutic angiogenesis in myocardial ischemia. BM‐derived EPCs (*C‐Kit* ^+^‐BMCs) had been used to treat liver cirrhosis. Yu et al [32] reported that both liver cirrhosis and hepatocellular carcinoma led to increased expression of proangiogenic factors, which resulted in the recruitment of EPCs into nonmalignant and cirrhotic liver tissues. Shirakura et al. [33] indicated the existence of impaired EPCs function and differentiation in BM‐derived EPCs in liver fibrosis mice. After MCD‐induced MASH injury, our previous study observed a key cluster of *C‐Kit* ^+^‐LSECs with a changed phenotype [11]. Crosby et al also identified a *C‐Kit* ^+^‐cell population with stem cell characteristics located in the hepatic portal area of adult cirrhotic and normal livers, and some cells were CD31^+^ [34]. So in this study, we selected BMT of *C‐Kit* ^+^‐BMCs model in vivo to validate the regulating mitochondrial function of Mertk in LSECs. Hence, we focused on whether *Mertk* of LSECs regulates PINK1 mediated mitophagy in MASH. However, mitochondria is key regulator of EC homeostasis by controlling signaling responses to the environment [35]. It has been demonstrated that reducing mitochondria‐derived ROS (mtROS) can ameliorate hepatic steatosis, substantiating the crucial role of mtROS in EC dysfunction [36, 37]. Hyperglycemia or hyperlipidemia cause mitochondrial dysfunction, including opening of permeability transition pores and release of proapoptotic cytokines, leading to mitochondrial and EC apoptosis [38, 39]. In our study, IF staining of COX4/LC3B, mtCMXRos and mtSOX revealed mitophagy and MMP damage but excessive mtROS production in steatotic LSECs overexpressing *Mertk*, especially those in the PINK1/Parkin pathway, was inhibited. Interestingly, *Mertk* suppression markedly rescued mitophagy through stimulating the PINK1/Parkin pathway in steatotic LSECs and in MASH mice. Thus, our data supports the vital role of mitochondrial impairment in MASH endotheliopathy. We hypothesize that *Mertk* knocking‐down in LSECs will improve mitophagy through the PINK1/Parkin pathway in MASH.

There are several limitations to our studies that are worth noting. First, MASH is a chronic liver disease that can be life‐threatening and take years to progress. Our study evaluates *Mertk*/PINK1 levels after 8 weeks of MCD feeding and 2 weeks following BMT, but it is unclear whether these effects persist over the long term. Hence, it is recommended to further validate the findings over a longer period. Second, while our study establishes the role of PINK1 and mitophagy in mitochondrial homeostasis, other key mitochondrial functions, such as oxygen consumption rate (OCR) and ATP production, also play a crucial role in this pathway. Assessing these parameters would provide a more comprehensive understanding of mitochondrial function in *Mertk*‐PINK1 regulation.

Finally, our study reveals a potential role of *Mertk*/p‐ERK negative regulation of PINK1‐mediated mitophagy in LSECs in MASH. However, we first suggest that transplanting of LSECs with Mertk knocking‐down has a therapeutic effect on restoring mitophagy and MASH. The data highlight the need for future investigation to fully elucidate its therapeutic potential in human MASH.

## Author Contributions

**Yu‐Xuan Gao:** investigation, writing – original draft, methodology, software, formal analysis. **Zhong Weng:** investigation, methodology, software, validation. **Long Tang:** investigation, writing – original draft, visualization, data curation, formal analysis. **Ming‐Yi Xu:** conceptualization, investigation, writing – review and editing, supervision, validation, funding acquisition. **Sheng‐Zheng Luo:** conceptualization, writing – review and editing, supervision, project administration.

## Conflicts of Interest

The authors declare no conflicts of interest.

## Supporting information


**Table S1:** Primers used in qPCR. **Table S2:** Antibodies used in Western Blot. **Table S3:** Antibodies used in IF. **Figure S1:** The expressions of Mertk/p‐Mertk in LSECs *in vitro*.

## Data Availability

Data sharing not applicable to this article as no datasets were generated or analyzed during the current study. The datasets generated during and/or analyzed during the current study are available from the corresponding author on reasonable request.
