# Supplementary material for: Mertk + Liver Sinusoidal Endothelial Cells Negatively Regulate PINK1 Related Mitophagy and Accelerate MASH
Source: Immun Inflamm Dis. 2025 Sep 18;13(9):e70256. doi: 10.1002/iid3.70256 (PMC12444409; doi:10.1002/iid3.70256)
Supplement: Supplementary file 1 — Table S1: Primers used in qPCR. Table S2: Antibodies used in Western Blot. Table S3: Antibodies used in IF. Figure S1: The expressions of Mertk/p‐Mertk in LSECs in vitro. [file IID3-13-e70256-s001.pdf]

Supplemental Tables

Table S1 Primers used in qPCR

Gene	species	Primer	Sequence (5'-3')	Product size
Mertk	Mouse	Forward	AAGGTCCCCGTCTGTCCTAA	221bp
		Reverse	GCGGGGAGGGGATTACTTTG	
Pink1	Mouse	Forward	CACACTGTTCCTCGTTATGAAGA	194bp
		Reverse	CTTGAGATCCCGATGGGCAAT	
Parkin	Mouse	Forward	ACAGGGCTCCTGACATCTG	192bp
		Reverse	CAAGGACACGTCGGTAGCTT	
LC3b	Mouse	Forward	AGCAGCATCCAACCAAAATC	215bp
		Reverse	CTGTGTCCGTTCACCAACAG	
P62	Mouse	Forward	TGAAAGAACGCGTGCTGATAC	192bp
		Reverse	ATTTGGGGTCTCCACCCTTG	
GAPDH	Human	Forward	TCAAGAAGGTGGTGAAGCAGG	115bp
		Reverse	TCAAAGGTGGAGGAGTGGGT	
Mertk	Human	Forward	GCCATTGAACTTACCTTACATAG	140bp
		Reverse	TTTCCTTCCATTACAGACCC	
ERK	Human	Forward	TACTATGAGAAGCAGGAGCTCAGT	204bp
		Reverse	CTCCTGGATGCTTGTCTGGTAA	
Pink1	Human	Forward	GCCTCATCGAGGAAAAACAGG	114bp
		Reverse	GTCTCGTGTCCAACGGGTC	
GAPDH	Mouse	Forward	CATCAAGAAGGTGGTGAAGC	205bp
		Reverse	TGACAAAGTGGTCGTTGAGG	

Table S2 Antibodies used in Western Blot

Antibody	Species	Manufacturer	Catalog #	Dilution
ERK	Rabbit	Affinity	AF0155	1:1000
p-ERK	Rabbit	Affinity	AF1015	1:1000
MERTK	Rabbit	ABclonal	A5443	1:500
p-MERTK	Rabbit	Affinity	AF8443	1:1000
GAPDH	Rabbit	Abcam	ab181602	1:1000
LC3B	Rabbit	Proteintech	14600-1-AP	1:1000
P62	Mouse	Proteintech	66184-1-Ig	1:5000
PARKIN	Rabbit	Affinity	AF0235	1:500
PINK1	Rabbit	Affinity	DF7742	1:1000
HRP Conjugated AffiniPure Goat Anti-Mouse IgG (H+L)	Goat	Boster	BA1050	1:10000
HRP Conjugated AffiniPure Goat Anti-Rabbit IgG (H+L)	Goat	Boster	BA1054	1:10000

Table S3 Antibodies used in IF

Antibody	Species	Manufacturer	Catalog #	Dilution
COX4	Mouse	Abcam	ab33985	1:1000
LC3B	Rabbit	Proteintech	14600-1-AP	1:500
MERTK	Rabbit	ABclonal	A5443	1:200
PINK1	Mouse	Santa Cruz	Sc-517353	1:100
488-conjugated Goat Anti-Mouse IgG (H+L)	Goat	ABclonal	AS037	1:200
594-conjugated Goat Anti-Rabbit IgG (H+L)	Goat	ABclonal	AS039	1:200

Supplementary Figures

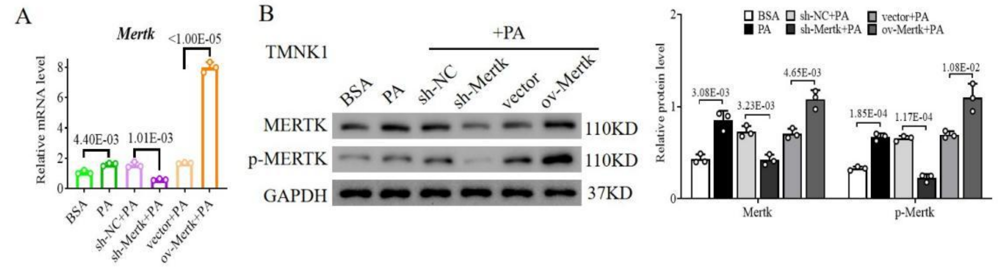

Figure S1 The expressions of Mertk/p-Mertk in LSECs *in vitro*

The TMNK-1 cells were divided into 6 groups: BSA, PA sh-NC+PA, sh-Mertk+PA, vector+PA and ov-Mertk+PA. The (A) mRNA and (B) protein levels of Mertk/p-Mertk were examined.

All the data are expressed as the mean±SEM. Two-sample Student's t tests were used for statistical analyses. *p* values indicate a significant difference compared to BSA/sh-NC+PA/ vector+PA-transfected TMNK-1 cells.

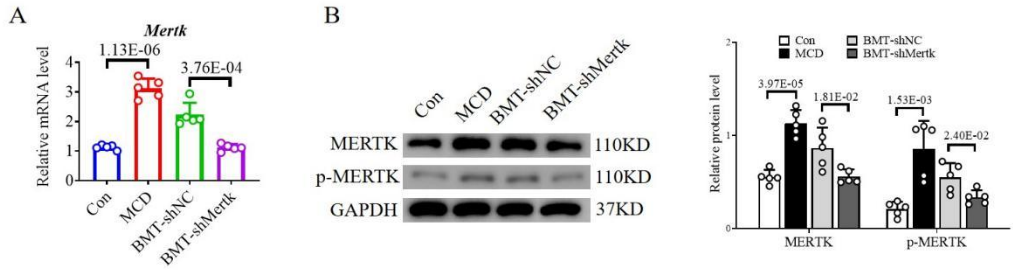

Figure S2 The expressions of Mertk/p-Mertk in BMT mice

The *in vivo* study was performed in 4 groups including Con, MCD, BMT-shNC and BMT-shMertk, each group n=5. The (A) mRNA and (B) protein levels of Mertk/p-Mertk were examined.

All the data are expressed as the mean±SEM. Two-sample Student's t tests were used for statistical analyses. *p* values indicate a significant difference compared to Con/BMT-Neg group.
